# Supplementary material for: Glucose-Binding of Periplasmic Protein GltB Activates GtrS-GltR Two-Component System in Pseudomonas aeruginosa
Source: Microorganisms. 2021 Feb 21;9(2):447. doi: 10.3390/microorganisms9020447 (PMC7927077; doi:10.3390/microorganisms9020447)
Supplement: Supplementary file 1 [file microorganisms-09-00447-s001.zip › microorganisms-1107194-supplementary- for proofreading/Supplementary Materials.docx]

**Supplementary Materials**

**Figures and Figure legends**


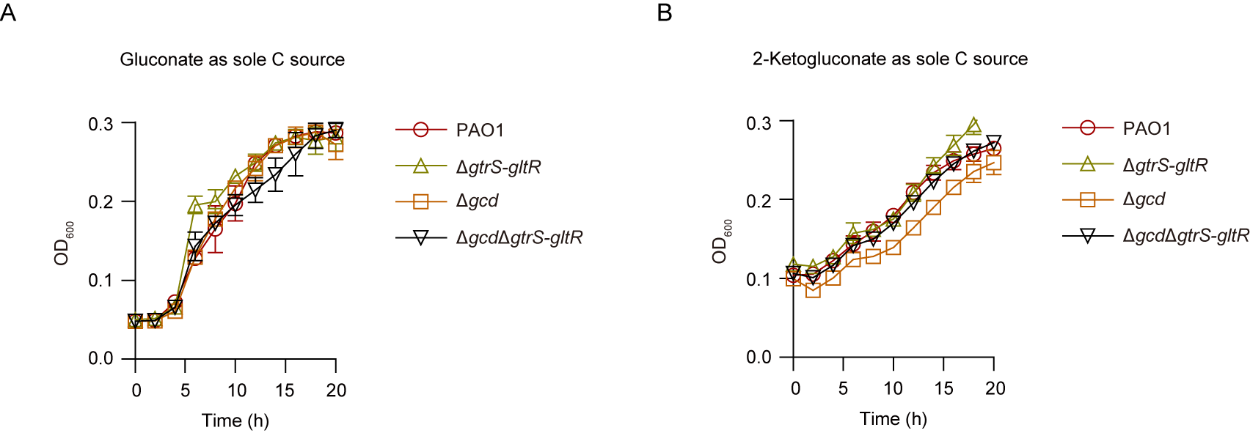


**Fig.S1. The growth curve of *P. aeruginosa* strains.** Bacteria were cultured in 96-well plates containing MM supplemented with either 10 mM gluconate (A) or 10 mM 2-ketogluconate (2-KG) (B) as the sole carbon source. OD_600_, an optical density at 600 nm. Data from n = 3 biological replicates reported as mean ± SD.


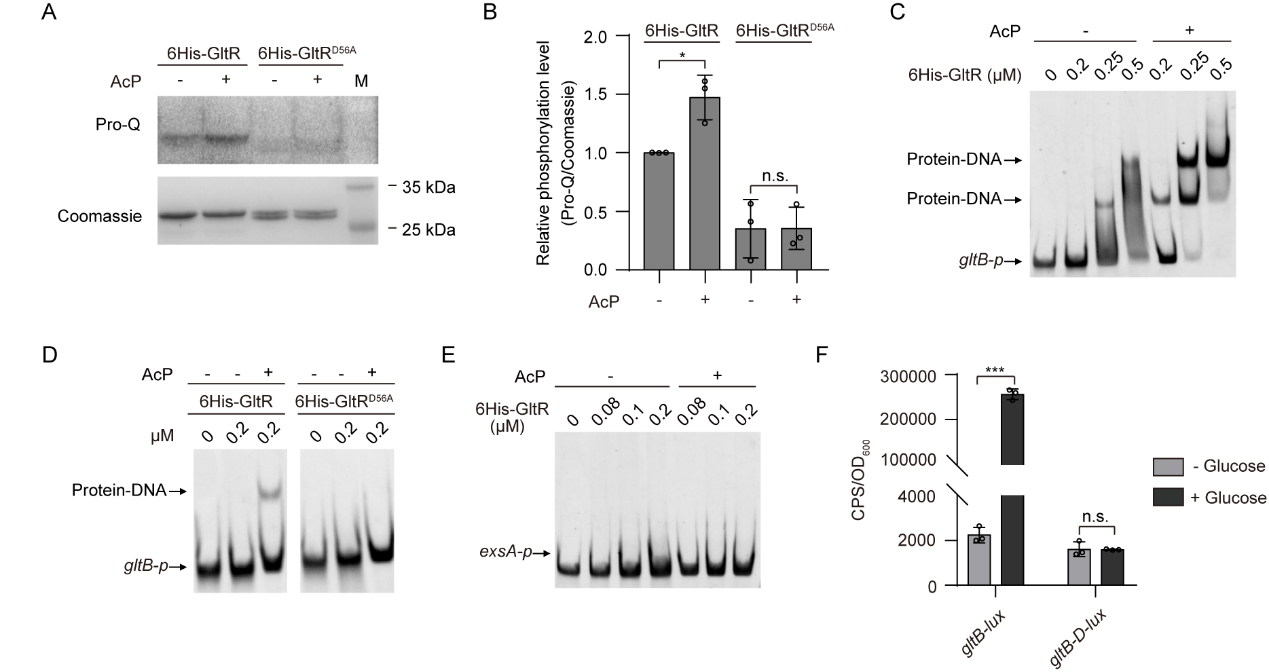


### Fig. S2. Phosphorylation assays, electrophoretic mobility shift assays, and promoter activity assays**.** (A and B) Representative images (A) and analysis (B) of the phosphorylation of 6His-GltR and 6His-GltR^D56A^ in the absence (−) or presence (+) of 50 mM acetyl phosphate (AcP) at 37°C for 30 min. The intensities of Pro-Q diamond stained bands of proteins were standardized against the intensities of the same bands after gels restained with Coomassie blue, and was further normalized to the vehicle group [6His-GltR in the absence (-) of AcP]. M, protein marker. In (B), data from n = 3 biological replicates reported as mean ± SD. (*P < 0.05, n.s. indicates no significant difference; Student's two-tailed *t*-test). (C) EMSA showing the binding of N-terminal His_6_-tagged GltR (i.e., 6His-GltR) to the promoter DNA of *gltB* (*gltB-p*) in the absence (-) or presence (+) of 50 mM acetyl phosphate (AcP). **(D)** EMSAs showing the binding of 6His-GltR or 6His-GltR^D56A^ to the promoter DNA of *gltB* (*gltB-p*) in the absence (−) or presence (+) of AcP (50 mM). **(E)** EMSA showing the binding of 6His-GltR to the promoter DNA of *exsA* (*exsA-p*) in the absence (-) or presence (+) of AcP (50 mM). (F) Role of the conserved GltR-binding site (i.e., 5’-GTGACAAA-3’) in the promoter activity of *gltB*. *P. aeruginosa* strains were grown in tubes containing M9 MM supplemented with (+) or without (-) 5 mM glucose at 37°C with shaking (250 rpm) for 8 h. *gltB-D-lux*, *gltB-lux* variants lacking the conserved GltR-binding site, 5’- GTGACAAA-3’. Data from n = 3 biological replicates reported as mean ± SD (***P < 0.001, n.s. indicates no significant difference; Student's two-tailed *t*-test).


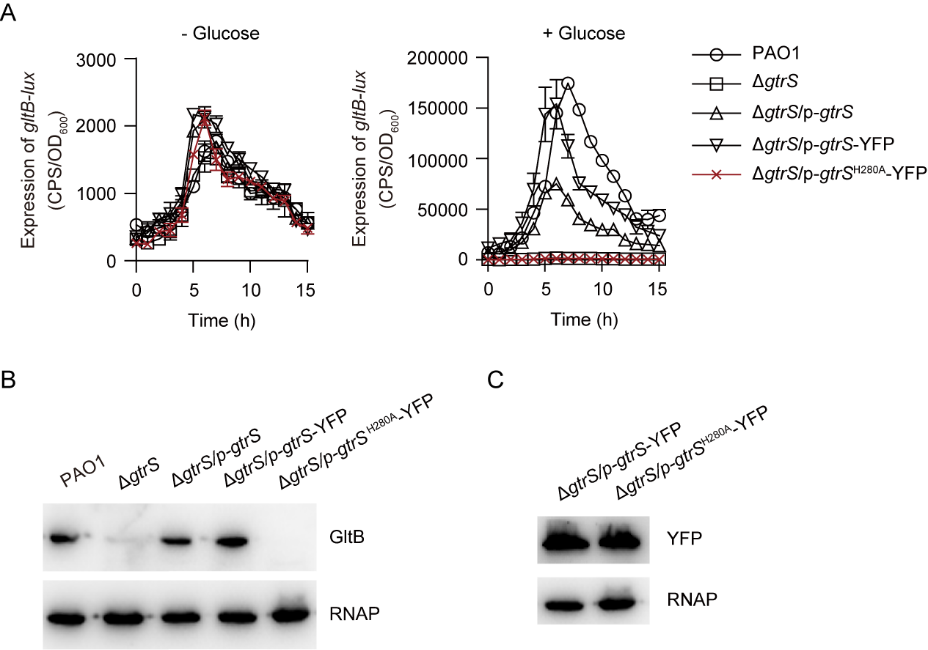


### Fig. S3. **Effect of H280A amino acid substituent in GtrS on the transcriptional regulatory activity of GtrS-GltR. (A)** The expression of *gltB-lux* in *P. aeruginosa* strains cultured in 96-well plates containing M8 MM supplemented with (+) or without (-) glucose (10 mM). CPS, counts per second; OD_600_, an optical density at 600 nm. Data from n = 3 biological replicates reported as mean ± SD. PAO1 and Δ*gtrS* harbor an empty pAK1900 vector as control; p-*gtrS* denotes the pAK1900-*gtrS* plasmid; p-*gtrS*-YFP denotes the pAK1900-*gtrS*-YFP plasmid; p-*gtrS*^H280A^-YFP denotes the pAK1900-*gtrS*^H280A^-YFP plasmid. (B) Western blot images showing the production of GltB. (C) Western blot images showing the production of GtrS or GtrS^H280A^ in Δ*gtrS* carrying indicated plasmid. In (B) and (C), protein samples were derived from bacteria grown in tubes containing M8 MM supplemented with 10 mM glucose at 37°C for 6 h with shaking (250 rpm). RNAP is probed as a loading control.


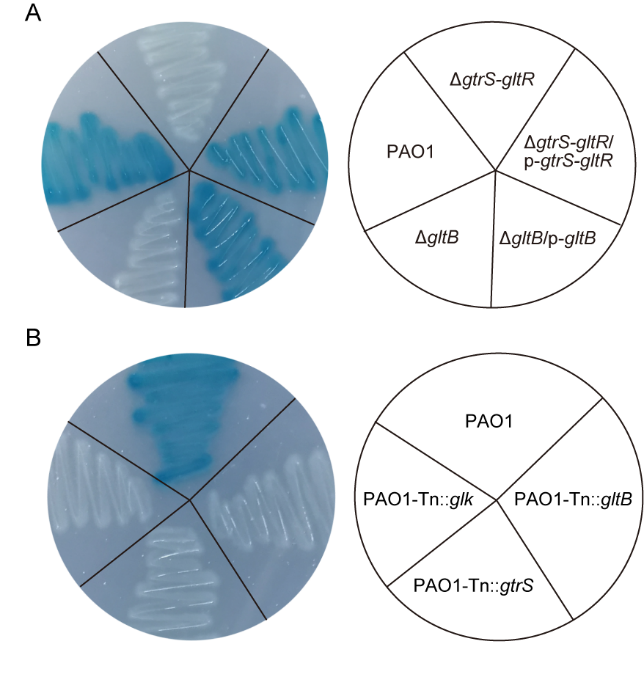


**Fig. S4. Effect of gene disruption in PAO1 on the expression of *gltB-lacZ*.** (A) PAO1, Δ*gltB*, and Δ*gtrS-gltR* harbor an empty pAK1900 vector as control; p-*gltB* denotes the pAK1900-*gltB* plasmid; p-*gtrS-gltR* denotes the pAK1900-*gtrS-gltR* plasmid. (B) PAO1-Tn::*gltB*, PAO1 strain with a transposon insertion in *gltB*; PAO1-Tn::*gtrS*, PAO1 strain with a transposon insertion in *gtrS*; PAO1-Tn::*glk*, PAO1 strain with a transposon insertion in *glk*. All strains harbor the mini-CTX-*gltB-lacZ* plasmid. In (A and B), strains grew on M8 minimal agar medium supplemented with 10 mM glucose and 20 µg/ml 5-bromo-4-chloro-3-indolyl-β-D-galactoside (X-gal) and incubated at 37°C o­vernight.


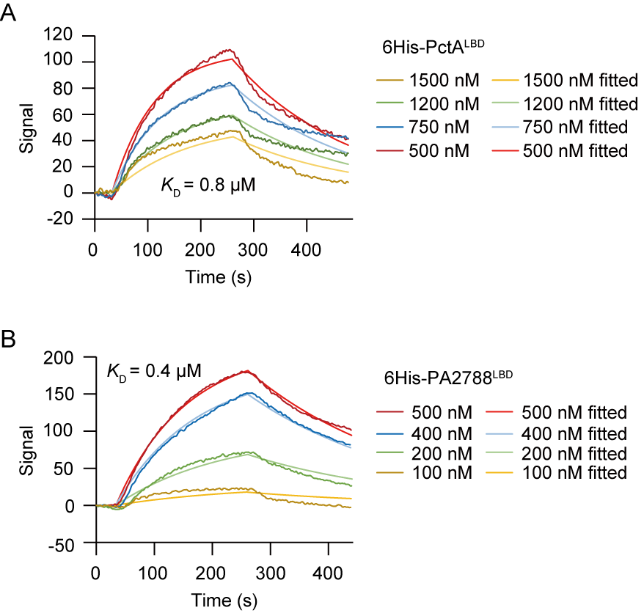


**Fig. S5.** **GltB interacts with chemotaxis proteins.** (A and B) Surface plasmon resonance (SPR) analysis showing the interaction of 6His-GltB with N-terminal His_6_-tagged ligand-binding domain of PctA (i.e., 6His-PctA^LBD^) (A) and N-terminal His_6_-tagged ligand-binding domain of PA2788 (i.e., 6His-2788^LBD^) (B). Analysis performed in TraceDrawer using a 1:1 binding interaction model. *K*_D_, equilibrium dissociation constant.


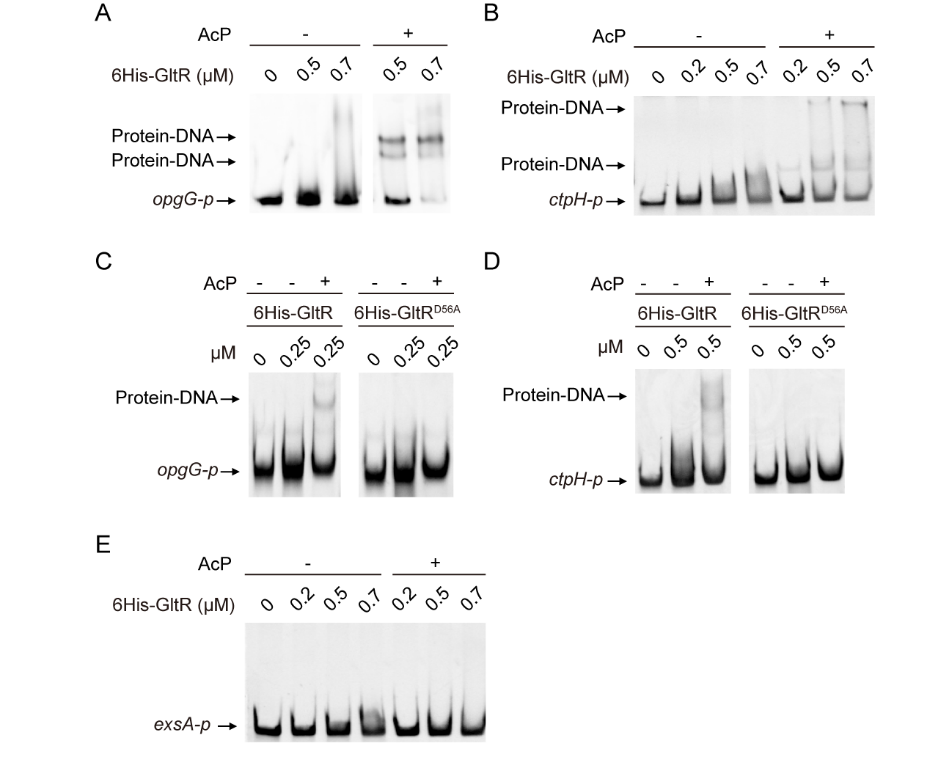


**Fig. S6.** **Electrophoretic mobility shift assays.** (A and B) EMSAs showing the binding of 6His-GltR to the promoter DNAs of *opgG* (*opgG-p*) (A) and *ctpH* (*ctpH­-p*) (B) in the absence (-) or presence (+) of AcP (50 mM). (C and D) Effect of D56A amino acid substituent on the binding ability of 6His-GltR to the promoter DNA of *opgG* (*opgG-p*) (C) and *ctpH* (*ctpH­-p*) (D) in the absence (−) or presence (+) of of AcP (50 mM). (E) EMSAs showing the binding of 6His-GltR to the promoter DNA of *exsA* (*exsA-p*) in the absence (-) or presence (+) of of AcP (50 mM).

**Table S1-S3**

**Table S1. Plasmids and bacterial strains and used in this study**

| Plasmids or strains | Relevant characteristics^a^ | Source |
| --- | --- | --- |
| **Plasmids** |  |  |
| pAK1900 | *E. coli–P. aeruginosa* shuttle cloning vector, Ap^r^ Cb^r^ | (1) |
| pEX18Ap | Gene replacement vector, mob^+^*sacB*, Ap^r^ | (2) |
| pPS858 | pBR322 derivative carrying a FRT-Gm cassette, Ap^r^ | (2) |
| pFLP2 | Source of Flp recombinase; Ap^r^/Cb^r^ | (2) |
| pET28a | T7 *lac* promoter–operator, N-terminal His tag, kan^r^ | Novagen |
| mini-CTX-lacZ | Gene delivery vector for inserting genes at the CTX phage *att* site on *P. aeruginosa* chromosome, Tc^r^; *lacZ-*based promoter reporter | (3) |
| mini-CTX-lux | Gene delivery vector for inserting genes at the CTX phage *att* site on *P. aeruginosa* chromosome, Tc^r^; *lux-*based promoter reporter | (3) |
| mini-*gltR-flag-lacZ* | mini-CTX-lacZ derivative carrying *gltR-flag* controlled by the *lac* promoter of the pAK1900 | This study |
| *gltB-lux* | mini-CTX-lux containing *gltB* promoter region (−740 to +20 of the start codon of *gltB*) | This study |
| mini-*gltB-lacZ* | mini-CTX-lacZ containing *gltB* promoter region (−740 to +20 of the start codon of *gltB*) | This study |
| *gltB-D-lux* | *gltB-lux* which lacking the conserved GltR-binding site, 5’-GTGACAAA-3’ | This study |
| p-*gtrS* | pAK1900 derivative carrying *gtrS* on a ~1.5 kb *Hin*dIII/*Bam*HI fragment in same orientation as p*lac* | This study |
| p-*gcd* | pAK1900 derivative carrying *gcd* on a ~2.4 kb *Hin*dIII/*Bam*HI fragment in same orientation as p*lac* | This study |
| p-*gtrS-gltR* | pAK1900 derivative carrying *gtrS-gltR* on a ~2.3 kb *Hin*dIII/*Bam*HI fragment in same orientation as p*lac* | This study |
| p-*gltB* | pAK1900 derivative carrying *gltB* on a ~1. 3 kb *Hin*dIII/*Kpn*I fragment in same orientation as p*lac* | This study |
| p-*gtrS-*YFP | pAK1900 derivative carrying a *gtrS-*YFP fusion gene for the expression of C-terminal YFP-tagged GtrS proteins | This study |
| p-*gtrS*^H280A^ | p-*gtrS* carrying *gtrS* which has alanine substitution mutant at the site of histidine 280 | This study |
| p-*gtrS*^H280A^-YFP | p-*gtrS-*YFP which has alanine substitution mutant at the site of histidine 280 | This study |
| p-*gltR*-flag | pAK1900 derivative carrying a *gltR*-flag fusion gene for the expression of C-terminal flag-tagged GltR proteins | This study |
| p-*gltB*^W33A^ | p-*gltB* carrying *gltB* which has alanine substitution mutant at the site of tryptophan 33 | This study |
| p-*gltB*^W34A^ | p-*gltB* carrying *gltB* which has alanine substitution mutant at the site of tryptophan 34 | This study |
| p-*gltB*^K90A^ | p-*gltB* carrying *gltB* which has alanine substitution mutant at the site of lysine 90 | This study |
| p-*gltB*^W268A^ | p-*gltB* carrying *gltB* which has alanine substitution mutant at the site of tryptophan 268 | This study |
| p-*gltB*^D301A^ | p-*gltB* carrying *gltB* which has alanine substitution mutant at the site of aspartate 301 | This study |
| pEX18Ap::*gltB*UGD | pEX18Ap derivative, for replacing *gltB* with a gentamicin resistance cassette from plasmid pPS858 | This study |
| pEX18Ap::*gtrS*UGD | pEX18Ap derivative, for replacing *gtrS* with a gentamicin resistance cassette from plasmid pPS858 | This study |
| pEX18Ap::*gtrS-gltR*UGD | pEX18Ap derivative, for replacing *gtrS-gltR* loci with a gentamicin resistance cassette from plasmid pPS858 | This study |
| pEX18Ap::*gad*UGD | pEX18Ap derivative, for replacing *gad*  with a gentamicin resistance cassette from plasmid pPS858 | This study |
| pEX18Ap::*gcd*UGD | pEX18Ap derivative, for replacing *gcd*  with a gentamicin resistance cassette from plasmid pPS858 | This study |
| pET28a-6His-*gltB* | pET28a derivative carrying *gltB* (*pa3190*) | This study |
| pET28a-6His-GltR | pET28a derivative carrying *gltR* (*pa3192*) | This study |
| pET28a-6His-GltR^D56A^ | pET28a derivative carrying *gltR* (*pa*3192) which has alanine substitution mutant at the site of aspartate 56 | This study |
| pET28a-GtrS^LBD^ | pET28a derivative carrying *gtrS*^LBD^ for the expression of an N-terminal His-tagged the ligand-binding domain of GtrS (residues 29-199) | This study |
| pET28a-PctA^LBD^ | pET28a derivative carrying *pctA*^LBD^ for the expression of an N-terminal His-tagged the ligand-binding domain of PctA (residues 30-278) | This study |
| pET28a-PA2788^LBD^ | pET28a derivative carrying *pa2788*^LBD^ for the expression of an N-terminal His-tagged the ligand-binding domain of PA2788 (residues 44-179) | This study |
| pET28a-6His-GltB^D301A^ | pET28a derivative carrying *gltB* which has alanine substitution mutant at the site of aspartate 301 | This study |
| pEYFP | plasmid containing YFP fragment | This study |
| p-*gtrS*-Y | p-*gtrS* without the stop codon | This study |
| pUT18C | A derivative of the high copy number vector pUC19 and encoding the T18 fragment (amino acids 225 to 399 of CyaA), Ap^r^ | This study |
| pKT25 | A derivative of the low copy number vector pSU40 and Encoding the T25 fragment (corresponding to the first 224 amino acids of CyaA), Kan^r^ | This study |
| pUT18C-zip | a derivative of pUT18C in which the leucine zipper of GCN4 is genetically fused in frame to the T18 fragment (inserted between the *Kpn*I and the *Eco*RI site of pUT18C) | This study |
| pKT25-zip | a derivative of pKT25 in which the leucine zipper of GCN4 is genetically fused in frame to the T25 fragment (inserted within the *Kpn*I site of pKT25) | This study |
| pUT18C*-*GltB | pUT18C containing GltB (lacking the first 25 codons encoding the N-terminal export signal) | This study |
| pKT25-GtrS^LBD^ | pKT25 containing the ligand-binding domain of GtrS (residues 29-199) | This study |
| *gntR-lux* | mini-CTX-lux containing *gntR* promoter region (−466 to +31 of the start codon of *gntR*) | This study |
| *ptxS-lux* | mini-CTX-lux containing *ptxS* promoter region (−749 to +27 of the start codon of *ptxS*) | This study |
| *glk-lux* | mini-CTX-lux containing *glk* promoter region (−730 to +56 of the start codon of *glk*) | This study |
| *edd-lux* | mini-CTX-lux containing *edd* promoter region (−520 to +26 of the start codon of *edd*) | This study |
| *toxA-lux* | mini-CTX-lux containing *toxA* promoter region (−980 to -5 of the start codon of *toxA*) | This study |
| *opgG-lux* | mini-CTX-lux containing *opgG* promoter region (−493 to +20 of the start codon of *opgG*) | This study |
| *ctpH-lux* | mini-CTX-lux containing *ctpH* promoter region (−229 to +99 of the start codon of *ctpH*) | This study |
| ***P. aeruginosa* strains** |  |  |
| PAO1 | Wild type | Lab stock |
| Δ*gltB* | PAO1 derivative with a gentamicin resistance cassette replaced the *gltB* gene | This study |
| Δ*gtrS* | PAO1 derivative with a gentamicin resistance cassette replaced the *gtrS* gene | This study |
| Δ*gtrS-gltR* | PAO1 derivative with a gentamicin resistance cassette replaced the *gtrS-gltR* locus | This study |
| Δ*gcd* | PAO1 derivative with a gentamicin resistance cassette replaced the *gcd* gene | This study |
| Δ*gad* | PAO1 derivative with a gentamicin resistance cassette replaced the *gad* gene | This study |
| Δ*gcd*Δ*gtrS-gltR* | PAO1 derivative with a gentamicin resistance cassette replaced the *gcd*, and a deletion of *gtrS-gltR* | This study |
| ***E. coli* strains** |  |  |
| DH5a | *endA hsdR17 supE44 thi-1 recA1 gyrA relA1*Δ(*lacZYA-argF*)*U169 deoR* (*φ80dlac*Δ(*lacZ*)*M15*) | Lab stock |
| BL21 | F^–^*ompT hsdS*_B_ (r_B_^-^ m_B_^-^) *gal dcm met* (DE3) | Lab stock |
| S17 λ-pir | *recA thi pro hsdR*^−^*M*^+^ RP4-2-Tc::Mu Km::Tn*7* λpir (Tp^r^ Str^r^) | Lab stock |
| S17 λ-pir (pFLP2) | S17 λ-pir containing pFLP2 | This study |
| SM10-λ *pir* (pBT20) | *E. coli* SM10-λ *pir* containing pBT20 | (4) |
| BTH101 | reporter strain for BACTH assay, Str^r^ | This study |

^a^Ap^r^, ampicillin resistance; Cb^r^, carbenicillin resistance; Kan^r^, kanamycin resistance; Tc^r^, tetracycline resistance; Tp^r^, trimethoprim resistance; Str^r^, Streptomycin resistance; Gm^r^, gentamycin resistance.

### Table S2. Primers used in this study.

| **Primer** | **Sequence** | **Purpose** |
| --- | --- | --- |
| AD2 | 5‘-CANGCTWSGTNTSCAA-3’ | For localization of the transposon |
| Gm447 | 5‘-GTGCAAGCAGATTACGGTGACGAT-3’ | For localization of the transposon |
| Gm464 | 5‘-TGACGATCCCGCAGTGGCTCTC-3’ | For localization of the transposon |
| Gm487 | 5‘-ATACAAAGTTGGGCATACG-3’ | For localization of the transposon |
| *gtrS*-comp-F  *gtrS*-comp-R | 5‘-CCCAAGCTTCAGTGGCTACCTGCTGG-3’  5‘-TTTGGATCCGCCAGCCCATCACTCCA-3’ | Construction of plasmids p-*gtrS* and p-*gtrS*-YFP  Construction of p-*gtrS* plasmid and p-*gtrS-gltR* plasmid |
| *gltR*-comp-F | 5‘-CCCAAGCTTCTGAGTCGGCGTCGAGCA-3’ | Construction of plasmids p-*gltR*-flag and p-*gtrS-gltR* |
| *gtrS*-Y-R | 5‘-TTTGGATCCCTCCAGCCCCAGGCGTG-3’ | Construction of p-*gtrS*-YFP plasmid |
| *gltB*-comp-F | 5‘-GGGAAGCTTGCGCAGACTTGCTCCGAA-3’ | Construction of plasmid p-*gltB* |
| *gltB*-comp-R | 5‘-TTTGGTACCATCCGCGCCAGGGACTTA-3’ | Construction of plasmid p-*gltB* |
| *gcd*-comp-F | 5‘-CCCAAGCTTTGTCATCAACCCCGAAGGGA-3’ | Construction of plasmid p-*gcd* |
| *gcd*-comp-R | 5‘-TTTGGATCCTGGGCGGGTCATTGCCTGGG-3’ | Construction of plasmid p-*gcd* |
| D-*gtrS*-up-F | 5‘-CCGGAATTCGCGCTTACCTGCAGGACGTA-3’ | Deletion of *gtrS* |
| D-*gtrS*-up-R | 5‘-CGCGGATCCTAGCTGACGCTGGCCGCAT-3’ | Deletion of *gtrS* |
| D-*gtrS*-down-F | 5‘-CGCGGATCCTGGCGGCGAAGTCAGTTTGC-3’ | Deletion of *gtrS* and *gtrS-gltR* |
| D-*gtrS*-down-R | 5‘-CCCAAGCTTGAGCCAGTTCACCCGGTGT-3’ | Deletion of *gtrS* and *gtrS-gltR* |
| D-*gltR*-up-F | 5‘-GGTGAATTCATGGGGTGAACGGCGAGTT-3’ | For deletion of *gtrS-gltR* |
| D-*gltR*-up-R | 5‘-TTTGGATCCCTCACGAGGCTCCTCCTTAT-3’ | For deletion of *gtrS-gltR* |
| D-*gltB*-up-F | 5‘-GGTGAATTCCCTTCAACACCATGCATG-3’ | Deletion of *gltB* |
| D-*gltB*-up-R | 5‘-TTTGGTACCGCGACGGATCGCATTCAT-3’ | Deletion of *gltB* |
| D-*gltB*-down-F | 5‘-GTTGGTACCCCAACTTCTTCAACGACC-3’ | Deletion of *gltB* |
| D-*gltB*-down-R | 5‘-TTTAAGCTTGACGCTGTCCCAGGCCTT-3’ | Deletion of *gltB* |
| D-*gcd*-up-F | 5‘-CCGGAATTCGCTTCCCCTGCGATTTCCAG-3’ | Deletion of *gcd* |
| D-*gcd*-up-R | 5‘-CGCGGATCCTTGATGCCGCCGGCCAGCA-3’ | Deletion of *gcd* |
| D-*gcd*-down-F | 5‘-CGCGGATCCGCAAGCAGCTCTGGCAGG -3’ | Deletion of *gcd* |
| D-*gcd*-down-R | 5‘-CCCAAGCTTATGCTCTTGCGCGTGTCGT-3’ | Deletion of *gcd* |
| D-*gad*-up-F | 5‘-CCGGAATTCTGCGCGGTAGACAACCTGCT-3’ | Deletion of *gad* |
| D-*gad*-up-R | 5‘-CGCGGATCCGCCTCGGTCAGCTCCTTGG-3’ | Deletion of *gad* |
| D-*gad*-down-F | 5‘-CGCGGATCCATCCGAAGACCAGTGTGCTC-3’ | Deletion of *gad* |
| D-*gad*-down-R | 5‘-CCCAAGCTTCCCGCAGTTGTCGACGTAGA-3’ | Deletion of *gad* |
| pAK1900-mini-F | 5‘-TGCTCTAGATATCACGAGGCCCTT-3’ | Generation of mini-*gltR*-flag |
| pAK1900-mini-R | 5‘-TGGTCTAGAACGGCCAGTGAATTG-3’ | Generation of mini-*gltR*-flag |
| *gltB*-F | 5‘-GGTCATATGATGAATGCGATCCGTCGCCT-3’ | Construction of pET28a-*gltB* plasmid |
| *gltB*-R | 5‘-TTTCTCGAGATCCGCGCCAGGGACTTA-3’ | Construction of pET28a-*gltB* plasmid |
| *gtrS*-LBD-F | 5‘-GGTCATATGCTGTCGCACCTGCGCT-3’ | Construction of pET28a-*gtrS*^LBD^ plasmid |
| *gtrS*-LBD-R | 5‘-TTTGGATCCTCAGAGCACCTGCTGC-3’ | Construction of pET28a-*gtrS*^LBD^ plasmid |
| *gltR*-F | 5‘-TTTGGATCCGTGAGCGCGAACGGACGATC-3’ | Construction of pET28a-*gltR* plasmid |
| *gltR*-R | 5‘-TTTCTCGAGAGCGCCGCCGTTCGCTCA-3’ | Construction of pET28a-*gltR* plasmid |
| *gltR*-D56A-F | 5’‑GCGAGCCTGGCGATCCTCGCGGTGATGCTGCCGGACGAGGAC-3´ | Construction of pET28a-*gltR*^D56A^ plasmid |
| *gltR*-D56A-R | 5´‑CTCGTCCGGCAGCATCACCGCGAGGATCGCCAGGCTCGCCTC-3´ | Construction of pET28a-*gltR*^D56A^ plasmid |
| *pctA*-LBD-F | 5´‑CGCGGATCCAACGATTACCTGCAGCGCAA-3´ | Construction of pET28a-*pctA*^LBD^ plasmid |
| *pctA*-LBD-R | 5´‑TTTAAGCTTTCAGGCCGAGACGCGGAACT-3´ | Construction of pET28a-*pctA*^LBD^ plasmid |
| *pa2788*-LBD-F | 5´‑CGCGGATCCTACCTGAGCATGTCGATCTC-3´ | Construction of pET28a-*pa2788*^LBD^ plasmid |
| *pa2788*-LBD-R | 5´‑CCCAAGCTTTCACCACATCTGGGTGTG-3´ | Construction of pET28a-*pa2788*^LBD^ plasmid |
| YFP-F | 5‘-TTTGGATCCCCGGTCGCCACCATGGTGAG-3’ | Construction of plasmids p-*gtrS-*YFP |
| YFP-R | 5‘-CGGGGTACCAGGCCTCTGCAGTCGACTTA-3’ | Construction of plasmids p-*gtrS-*YFP |
| *gltR*-flag-R | 5‘-CGCGGATCCTTACTTATCGTCGTCATCCTTGTAATCTGGCTGCAGGTGCGGC-3’ | Construction of p-*gltR*-flag |
| GtrS (H280A)-F | 5‘-TGTTCAGCGCCATCTCCGCCGACCTGCGCA-3’ | Generation of p-*gtrS*^H280A^ and p-*gtrS*^H280A^-YFP |
| GtrS (H280A)-R | 5‘-TGCGCAGGTCGGCGGAGATGGCGCTGAACA-3’ | Generation of p-*gtrS*^H280A^ and p-*gtrS*^H280A^-YFP |
| pro-*gltB*-F | 5‘-TTTCTCGAGCACGTCGACGACGAC-3’ | Construction of mini-*gltB-lux* and mini-*gltB-lacZ* |
| pro-*gltB*-R | 5‘-TTTGGATCCAGGCGACGGATCGCA-3’ | Construction of mini-*gltB-lux* and mini-*gltB-lacZ* |
| pro-*gltB-D*-F | 5‘-CCCAATGTAACCGCTCCCCCGGATCGCTTCGTTAC-3’ | Construction of mini-*gltB-D-lux* |
| pro-*gltB-D*-R | 5‘-GTAACGAAGCGATCCGGGGGAGCGGTTACATTGGG-3’ | Construction of mini-*gltB-D-lux* |
| pro-*gntR*-F | 5‘-TTTAAGCTTTAGGCGCGCTTGAGGGCGGAA-3’ | Construction of mini-*gntR-lux* |
| pro-*gntR*-R | 5‘-CGCGGATCCGCGTGTTCTTGTCGTTCTTG-3’ | Construction of mini-*gntR-lux* |
| pro-*ptxS*-F | 5‘-TTTAAGCTTGGGTGGTACGGGTCAGCAAC-3’ | Construction of mini-*ptxS-lux* |
| pro-*ptxS*-R | 5‘-GGTGAATTCACGGCTGGGCAGTACTGAAC-3’ | Construction of mini-*ptxS-lux* |
| pro-*glK*-F | 5‘-CCCAAGCTTGAGTCTCGACGAAGC-3’ | Construction of mini-*glK-lux* |
| pro-*glK*-R | 5‘-TTTGGATCCCCGATATCGCCGACCA-3’ | Construction of mini-*glK-lux* |
| pro-*edd*-F | 5‘-CCCAAGCTTCTTCGACATCCTTGCC-3’ | Construction of mini-*edd-lux* |
| pro-*edd*-R | 5‘-TTTGGATCCGTGACTTCGAGCACAC-3’ | Construction of mini-*edd-lux* |
| pro-*toxA*-F | 5‘-CCCAAGCTTATATCCCGTGTTTAGA-3’ | Construction of mini-*toxA-lux* |
| pro-*toxA*-R | 5‘-TTTGGATCCATGGCTCCTTTGATGG-3’ | Construction of mini-*toxA-lux* |
| pro-*opgG*-F | 5‘-TTTAAGCTTCAACGACGGGCCGGTGACCT-3’ | Construction of mini-*opgG-lux* |
| pro-*opgG*-R | 5‘-CGCGGATCCGAAACGGAACGGAAAATCACG-3’ | Construction of mini-*opgG-lux* |
| pro-*ctpH*-F | 5‘-CCCAAGCTTTCAACGAGTGCAACTGGCGG-3’ | Construction of mini-*ctpH-lux* |
| pro-*ctpH*-R | 5‘-TTTGGATCCTTGCAGCATGGAACGCCTCG-3’ | Construction of mini-*ctpH-lux* |
| GltB (W33A)-F | 5‘-CGAAGTCGAAGTGCTGCACGCGTGGACCTCCGCCG-3’ | Construction of p-*gltB*^W33A^ |
| GltB (W33A)-R | 5‘-CGGCGGAGGTCCACGCGTGCAGCACTTCGACTTCG-3’ | Construction of p-*gltB*^W33A^ |
| GltB (W34A)-F | 5‘-GAAGTCGAAGTGCTGCACTGGGCGACCTCCGCCGG-3’ | Construction of p-*gltB*^W34A^ |
| GltB (W34A)-R | 5‘-CCGGCGGAGGTCGCCCAGTGCAGCACTTCGACTTC-3’ | Construction of p-*gltB*^W34A^ |
| GltB (K90A)-F | 5‘-CGGCCGCGGCGCAGATCGCGGGGCCGGATATCCAG-3’ | Construction of p-*gltB*^K90A^ |
| GltB (K90A)-R | 5‘-CTGGATATCCGGCCCCGCGATCTGCGCCGCGGCCG-3’ | Construction of p-*gltB*^K90A^ |
| GltB (W268A)-F | 5‘-GCAGATCATGGGCGACGCGGCGAAGAGCGAGTTCA-3’ | Construction of p-*gltB*^W268A^ |
| GltB (W268A)-R | 5‘-TGAACTCGCTCTTCGCCGCGTCGCCCATGATCTGC-3’ | Construction of p-*gltB*^W268A^ |
| GltB (D301A)-F | 5‘-GCCTTCGACTACAACATCGCCTCGCTGGTGATGTT-3’ | Construction of p-*gltB*^D301A^ |
| GltB (D301A)-R | 5‘-AACATCACCAGCGAGGCGATGTTGTAGTCGAAGGC-3’ | Construction of p-*gltB*^D301A^ |
| *gltB*-pUT-F | 5‘-GCGTCTAGAGGGCGAAGTCGAAGTGCTGCACT-3’ | Generation of pUT18C-GltB plasmid |
| *gltB*-pUT-R | 5‘-TTTGGTACCTTACTGCGCCGCCGCCTCGATG-3’ | Generation of pUT18C-GltB plasmid |
| *gtrS*-LBD-pKT-F | 5‘-TTTTCTAGAGATGCTGTCGCACCTGCGC-3’ | Generation of pKT25-GtrS^LBD^ plasmid |
| *gtrS*-LBD-pKT-R | 5‘-TTTGGTACCTTCAAGAGCACCTGCTGC-3’ | Generation of pKT25-GtrS^LBD^ plasmid |
| pKT25-Bacth-F | 5‘-GTTCGCCATTATGCCGCATC-3’ | Sequencing of pKT25-GtrS^LBD^ plasmid |
| pKT25-Bacth-R | 5‘-GGATGTGCTGCAAGGCGATT-3’ | Sequencing of pKT25-GtrS^LBD^ plasmid |
| pUT18C-Bacth-F | 5‘-CGTCGCTGGGCGCAGTGGAACGCC-3’ | Sequencing of pUT18C-GltB plasmid |
| pUT18C-Bacth-R | 5‘-CTTAACTATGCGGCATCAGAGC-3’ | Sequencing of pUT18C-GltB plasmid |
| *exsA*-F (EMSA) | 5‘-GAATTGTACGGCCG-3’ | EMSA of *exsA* |
| *exsA*-R (EMSA) | 5‘-CTGCTTTCGGCCAA-3’ | EMSA of *exsA* |
| *gltB*-F (EMSA) | 5‘-GGTCCAGGGTACTGCGTTTC-3’ | EMSA of *gltB* |
| *gltB*-R (EMSA) | 5‘-ACAGATGACGGCAGAGAGGC-3’ | EMSA, footprint of *gltB* |
| *opgG*-F (EMSA) | 5‘-CAACGACGGGCCGGTGACCT-3’ | EMSA of *opgG* |
| *opgG*-R (EMSA) | 5‘-GAAACGGAACGGAAAATCACG-3’ | EMSA, footprint of *opgG* |
| *ctpH*-F (EMSA) | 5‘-TCAACGAGTGCAACTGGCGG-3’ | EMSA of *ctpH* |
| *ctpH*-R (EMSA) | 5‘-TTGCAGCATGGAACGCCTCG-3’ | EMSA, footprint of *ctpH* |
| *gltB*-F(EMSA)-FAM | 5‘-GGTCCAGGGTACTGCGTTTC-3’ | footprint of *gltB* |
| *opgG*-F(EMSA)-FAM | 5‘-CAACGACGGGCCGGTGACCT-3’ | footprint of *opgG* |
| *ctpH*-F (EMSA)-FAM | 5‘-TCAACGAGTGCAACTGGCGG-3’ | footprint of *ctpH* |

**Table S3. Transposon insertion sites**

| Gene | Insertion site^a^ | Description |
| --- | --- | --- |
| *pa*3190 (*gltB*) | 3580496(F)  3580496(R) 3581119(R)  3581208(R)  3581208(F)  3581228(R)^2^  3581246(R)^3^  3581996(R) | probable binding protein component of ABC sugar transporter |
| *pa*3191 (*gtrS*) | 3582384(R)  3582386(R)  3582390(R)^4^  3583066(F)  3584012(R) | glucose transport sensor, GtrS |
| *pa*3192 (*gltR*) | 3583807(R) 3583937(F) | two-component response regulator, GltR |
| *pa*3193 (*glk*) | 3585239(R) 3585263(R)  3585389(R) | glucokinase |

^a^ The mariner-based transposon (gentamicin resistance cassette) direction (F, forward; R, reverse.) relative to the genomic scaffold is provided. Number in top right corner represents times.

**References**

1. Jansons I*, et al.* (1994) Deletion and transposon mutagenesis and sequence analysis of the pRO1600 OriR region found in the broad-host-range plasmids of the pQF series. *Plasmid* 31(3):265-274.

2. Hoang TT, Karkhoff-Schweizer RR, Kutchma AJ, & Schweizer HP (1998) A broad-host-range Flp-FRT recombination system for site-specific excision of chromosomally-located DNA sequences: application for isolation of unmarked Pseudomonas aeruginosa mutants. *Gene* 212(1):77-86.

3. Becher A & Schweizer HP (2000) Integration-proficient Pseudomonas aeruginosa vectors for isolation of single-copy chromosomal lacZ and lux gene fusions. *BioTechniques* 29(5):948-950, 952.

4. Kulasekara HD*, et al.* (2005) A novel two-component system controls the expression of Pseudomonas aeruginosa fimbrial cup genes. *Molecular microbiology* 55(2):368-380.
